# Per Protocol Analysis and Long‐Term Outcomes of Laparoscopic Radical Resection for Gallbladder Cancer Compared With Open Method

**DOI:** 10.1002/wjs.70535

**Published:** 2026-08-24

**Authors:** Tae Ho Hong, Kwang Yeol Paik, Yoon kyung Woo, Jae Hyun Han, Jung Woo Lee, Jong Woo Lee, Dong Do You

**Affiliations:** ^1^ Department of Surgery Seoul St. Mary's Hospital College of Medicine The Catholic University of Korea Seoul Republic of Korea; ^2^ Department of Surgery Yeouido St. Mary's Hospital College of Medicine The Catholic University of Korea Seoul Republic of Korea; ^3^ Department of Surgery St. Vincent's Hospital College of Medicine The Catholic University of Korea Seoul Republic of Korea; ^4^ Department of Surgery Hallym University College of Medicine Hallym University Sacred Heart Hospital Anyang‐si South Korea; ^5^ Department of Surgery Gangneung Asan Medical Center University of Ulsan College of Medicine Ulsan South Korea

**Keywords:** gallbladder cancer, IVb/V bisegmentectomy, laparoscopic radical cholecystectomy, lymph node dissection, radical resection, wedge hepatic resection

## Abstract

**Background:**

Although laparoscopic radical resection for gallbladder (GB) cancer has been attempted, only a limited number of studies have evaluated its outcomes using a per‐protocol analytical approach.

**Methods:**

Between January 2010 and December 2019, 213 patients underwent surgery for gallbladder (GB) cancer at three institutions, of whom 203 consecutive patients with T2 or T3 disease were retrospectively analyzed. Protocol‐adherent radical resection (per‐protocol) was defined as wedge liver resection or IVb/V bisegmentectomy with the procurement of at least four lymph nodes. Propensity score matching was performed to compare the laparoscopic and open approaches.

**Results:**

Protocol‐adherent radical resection was achieved in 14 of 76 patients (18.4%) in the laparoscopic group and in 76 of 127 patients (59.8%) in the open surgery group. The primary unmet criterion for radical resection was an insufficient lymph node yield (60/76, 78.9%) in the laparoscopic group. After 1:1 propensity score matching (54 pairs), the 5‐year overall survival (OS) rate did not differ between the laparoscopic and open approaches (46.1% vs. 49.9%; hazard ratio 0.90, 95% confidence interval 0.55–1.47; *p* = 0.668), whereas the disparity in oncological adequacy persisted (protocol‐adherent radical resection 20.4% vs. 70.4%). Among patients who achieved protocol‐adherent radical resection (*N* = 90), the exploratory comparison of 5‐year OS between the laparoscopic (*N* = 14) and open (*N* = 76) groups showed no significant difference (61.5% vs. 53.3%; *p* = 0.288). Multivariate analysis in patients with protocol‐adherent radical resection identified N stage and elevated CA 19‐9 levels as independent prognostic factors for OS.

**Conclusions:**

Regardless of whether a laparoscopic or open approach is used, radical surgery—including appropriate hepatic resection with the retrieval of at least four lymph nodes—is essential for improving long‐term prognosis in patients with GB cancer. N stage and elevated CA 19‐9 levels were independently associated with overall survival.

## Graphical abstract

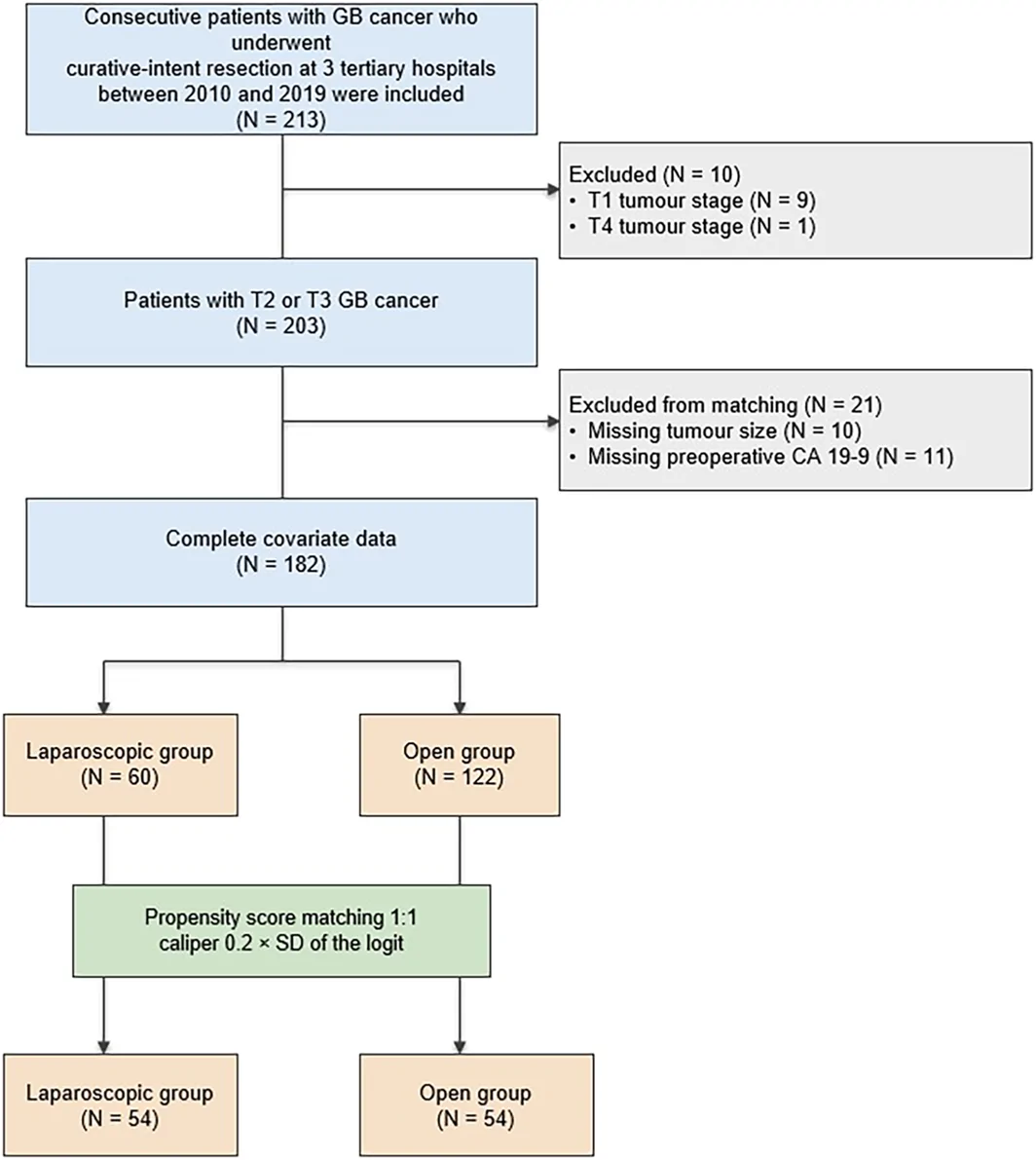

## 1 Introduction

Gallbladder (GB) cancer is a rare malignancy characterized by aggressive behavior, with radical resection representing the only curative treatment option. Multiple international guidelines have recommended that, except for T1a tumors (in which invasion is confined to the mucosal lamina propria), patients with T1b or more advanced tumors should undergo radical cholecystectomy. This procedure consists of wedge liver resection or IVb/V bisegmentectomy combined with locoregional lymphadenectomy and is considered the standard surgical therapy [1, 2, 3, 4].

In the era of minimally invasive surgery, the laparoscopic approach has been widely adopted for various gastrointestinal malignancies, and its role in the surgical treatment of GB cancer has been suggested to be feasible [5, 6, 7]. Laparoscopic surgery has been associated with reduced intraoperative blood loss and shorter hospital stays compared with open surgery. Short‐term outcomes, including postoperative morbidity and mortality, R0 resection rates, and port‐site and peritoneal recurrence rates, have been reported as acceptable [8, 9, 10, 11, 12]. However, long‐term outcomes with respect to overall survival have been reported in only a limited number of studies. Moreover, several retrospective studies have been constrained by relatively small sample sizes or short follow‐up periods between surgery and final assessment.

The present study evaluated long‐term outcomes following surgical resection of histopathologically confirmed GB cancer by comparing laparoscopic and open surgical approaches, with a follow‐up period of 52 months between surgery and medical record review. In addition, we re‐evaluated and performed quality control of laparoscopic and open radical resections for GB cancer, including hepatic resection and lymph node dissection, with specific attention to the number of harvested lymph nodes.

## 2 Patients and Methods

### 2.1 Study Cohort and Clinicopathological Variables

This study was approved by the Institutional Review Boards of the Catholic University of Korea and Hallym University. Between January 2010 and December 2019, 213 consecutive patients who underwent surgery for GB cancer at Seoul St. Mary's Hospital, St. Vincent's Hospital, and Hallym University Sacred Heart Hospital were retrospectively analyzed, with follow‐up through April 2024. A subset of patients from the first two centers overlaps with a previously published cohort [13] that did not evaluate surgical approach; the present study adds a third institution and addresses a distinct question using a different analytical framework.

After re‐review of operative records and pathological slides, 203 patients with T2 or T3 GB cancer were analyzed; 2 patients with T1a GB cancer, 7 with T1b GB cancer, and one with T4 GB cancer were excluded. All pathological slides were independently re‐reviewed by pathologists at each participating institution. Tumor staging was determined according to the eighth edition of the AJCC staging system [14].

The exclusion criteria were the presence of distant metastasis or peritoneal seeding, adenosquamous or neuroendocrine histology, and unexpected advanced disease identified intraoperatively. All clinicopathological data were prospectively recorded in a computerized database.

Surgery was undertaken only when curative resection appeared feasible on preoperative imaging. When intraoperative frozen section confirmed malignancy, concomitant liver resection with negative margins, regional lymphadenectomy and, if required, additional organ resections were performed to achieve R0 resection. The cystic duct margin was routinely examined by frozen section, and bile duct resection was added when the margin was suspicious or positive. For cancers diagnosed incidentally after simple cholecystectomy, additional resection was based on invasion depth, margin status and nodal risk. The extent of liver resection followed intraoperative findings and surgeon preference. Regional lymphadenectomy encompassed the cystic duct and common hepatic duct nodes, the hepatoduodenal ligament, and the posterior superior pancreatoduodenal nodes.

All surgical techniques described above were also attempted using a laparoscopic approach, depending on the surgeon's experience and preference.

Postoperatively, patients were followed at 3‐ to 6‐month intervals for tumor recurrence using CT imaging, carcinoembryonic antigen and carbohydrate antigen 19–9 (CA 19–9) assays and, in selected cases, positron emission tomography. Tumor recurrence was determined radiographically, and palliative chemotherapy or radiotherapy was administered when appropriate.

### 2.2 Definitions

According to the Practical Guidelines for the Surgical Treatment of Gallbladder Cancer, extended cholecystectomy—involving wedge resection of the GB bed or bisegmentectomy of liver segments IVb/V combined with regional lymph node dissection—is recommended for patients with T2 or T3 GB cancer. In the eighth edition of the AJCC staging system, N2 disease is defined as metastasis to more than four lymph nodes, and the IHPBA and the APHPBA International Study Group on Gallbladder Cancer recommend that a minimum of 4–6 lymph nodes be harvested for accurate staging [1, 2, 14]. In the present study, protocol‐adherent radical resection was defined as liver resection (either wedge resection or IVb/V bisegmentectomy) with retrieval of at least four lymph nodes. Resection was considered nonradical when liver resection was not performed or when three or fewer lymph nodes were retrieved.

### 2.3 Statistical Analysis

Descriptive statistics were presented as medians with ranges or as frequencies, as appropriate. Comparisons between groups were performed using the Mann‐Whitney *U* test, the chi‐square test, or Fisher's exact test. Overall survival (OS) was the primary endpoint and was estimated using the Kaplan‐Meier method. Survival differences between groups were assessed using the log‐rank test. To identify prognostic factors, multivariate analysis was conducted using Cox proportional hazards regression, incorporating all variables that showed a significant association with OS in univariate analysis (*p* < 0.10). Statistical significance was set at *p* < 0.05. All analyses were performed using SPSS software (version 22.0; IBM Corp., Armonk, NY, USA).

To reduce selection bias from the nonrandomized choice of approach, 1:1 propensity score matching was performed. Propensity scores were estimated by logistic regression on age, sex, T stage, tumor size and log‐transformed preoperative CA 19–9. Quality‐of‐resection variables (extent of hepatic resection, nodal yield, radical‐resection status) were deliberately excluded, being consequences of the approach and outcomes of interest. Nearest‐neighbor matching without replacement used a caliper of 0.2 standard deviations of the logit of the propensity score. Balance was assessed by standardized mean differences (adequate if < 0.1), which, unlike *p* values, are insensitive to sample size. Ten patients with unrecorded tumor size were excluded. Two sensitivity analyses were prespecified: matching that additionally included N stage, and a Cox model within the matched cohort adjusted for institution.

## 3 Results

### 3.1 Clinicopathological Characteristics

Of the 203 patients, 138, 48, and 17 patients were treated at Seoul St. Mary's, St. Vincent's, and Hallym University Sacred Heart Hospital, respectively. The median age of the entire cohort was 71 years (range, 36–94 years), and 95 patients (47%) were male. The T stage was T2a in 75 (37%), T2b in 54 (27%), and T3 in 74 (34%). Figure 1 summarizes the process of propensity score matching. The baseline clinicopathological characteristics before and after propensity score matching according to operative method are presented in Table 1.

**FIGURE 1:**
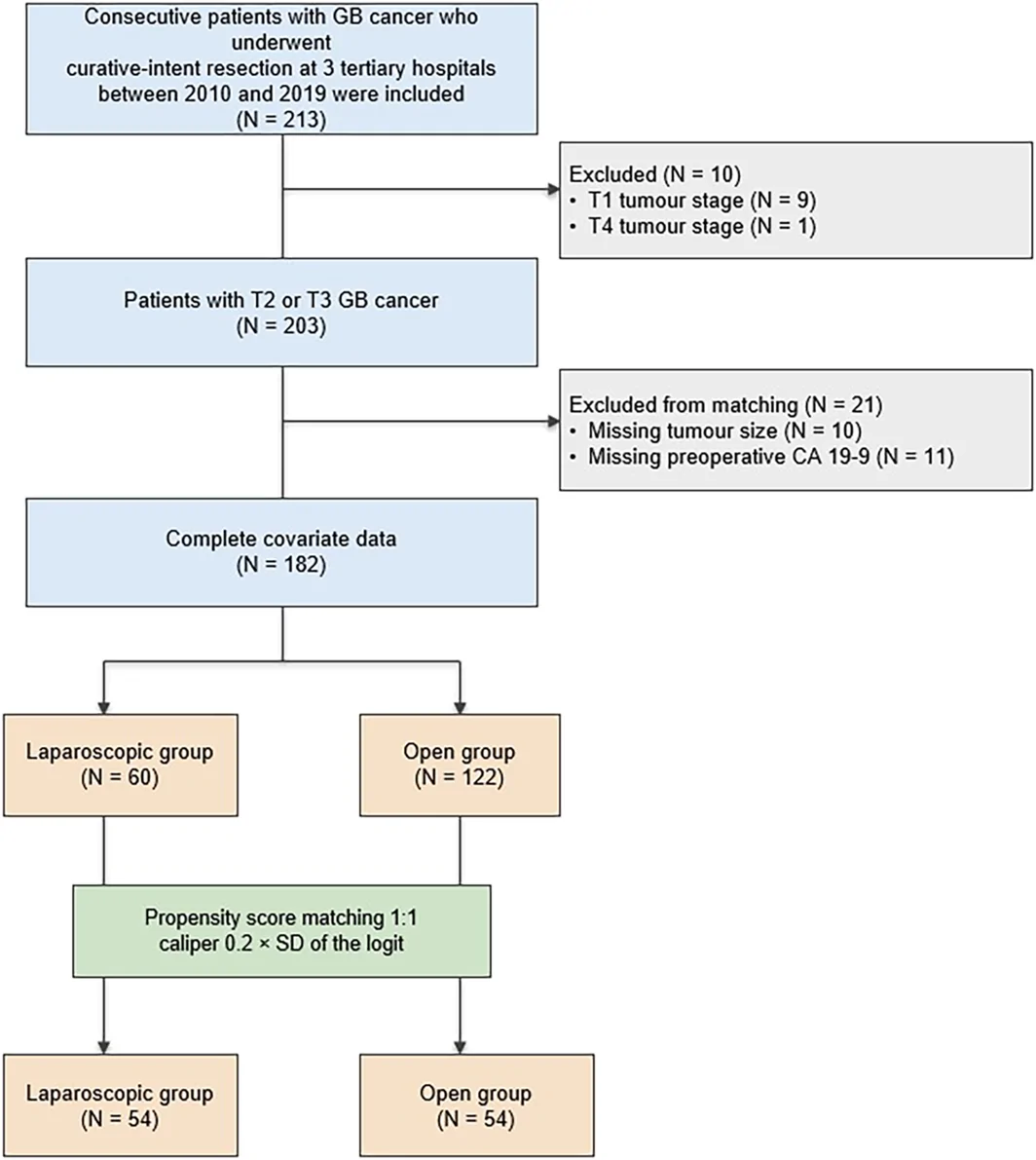
Patient selection and propensity score matching. GB, gallbladder; SD, standard deviation.

**TABLE 1:** Baseline and operative characteristics before and after propensity score matching.

Characteristic	Before matching				After matching			
	Lap (*n* = 76)	Open (*n* = 127)	*p*	SMD	Lap (*n* = 54)	Open (*n* = 54)	*p*	SMD
*Covariates included in the propensity model*								
Age, years	72.0 [46.0–94.0]	69.0 [36.0–86.0]	0.004	0.441	71.5 [46.0–94.0]	71.0 [52.0–83.0]	0.905	0.002
Male sex	37 (48.7%)	58 (45.7%)	0.677	0.060	24 (44.4%)	24 (44.4%)	1.000	0.000
T stage			0.098				0.971	
T2a	34 (44.7%)	41 (32.3%)		0.258	20 (37.0%)	20 (37.0%)		0.000
T2b	21 (27.6%)	33 (26.0%)		0.037	16 (29.6%)	17 (31.5%)		0.040
T3	21 (27.6%)	53 (41.7%)		0.300	18 (33.3%)	17 (31.5%)		0.040
Tumor size, cm	3.2 [0.4–8.8]	4.3 [0.9–14.2]	< 0.001	0.517	3.5 [0.4–8.8]	3.4 [0.3–7.5]	0.590	0.044
Preoperative CA 19–9, U/mL	130.4 ± 785.7	284.2 ± 1423.5	0.009	0.134	164.4 ± 886.9	41.7 ± 111.8	0.829	0.194
*Variables not included in matching*								
N stage			0.009				0.253	
N0	54 (71.1%)	68 (53.5%)		0.367	35 (64.8%)	33 (61.1%)		0.077
N1	18 (23.7%)	43 (33.9%)		0.226	17 (31.5%)	16 (29.6%)		0.040
N2	2 (2.6%)	16 (12.6%)		0.383	1 (1.9%)	5 (9.3%)		0.328
Liver resection			< 0.001				0.069	
Wedge	42 (55.3%)	74 (58.3%)		0.061	31 (57.4%)	33 (61.1%)		0.075
Segment IVb/V	12 (15.8%)	41 (32.3%)		0.393	11 (20.4%)	17 (31.5%)		0.256
No liver resection	22 (28.9%)	12 (9.4%)		0.511	12 (22.2%)	4 (7.4%)		0.426
Harvested lymph nodes ≥ 4	16 (21.1%)	81 (63.8%)	< 0.001	0.959	12 (22.2%)	40 (74.1%)	< 0.001	1.214
Protocol‐adherent radical resection	14 (18.4%)	76 (59.8%)	< 0.001	0.937	11 (20.4%)	38 (70.4%)	< 0.001	1.161
Operation time, min	102.5 ± 77.7	189.0 ± 90.1	< 0.001	1.029	113.9 ± 81.8	175.6 ± 89.8	< 0.001	0.719
Estimated blood loss, mL	100.0 [2–2000]	400.0 [2–3000]	< 0.001	0.679	100.0 [0.0–2000.0]	300.0 [0.0–1500.0]	0.005	0.466
Intraoperative transfusion	14 (18.4%)	37 (29.1%)	0.110	0.254	12 (22.2%)	11 (20.4%)	0.814	0.045
Adjuvant therapy	40 (52.6%)	89 (70.1%)	0.012	0.364	32 (59.3%)	35 (64.8%)	0.552	0.115

*Note:* For multi‐level variables a single overall *p* value is given. Values are compared between groups without pairing, as is conventional.

Abbreviation: SMD, standardized mean difference.

In the laparoscopic group, the absence of liver resection, retrieval of three or fewer lymph nodes, and nonradical resection were significantly more frequent than in the open group. Protocol‐adherent radical resection was achieved in 14 of 76 patients (18.4%) treated laparoscopically and in 76 of 127 (59.8%) treated openly. Liver resection was omitted in 22 of 76 (28.9%) versus 12 of 127 (9.4%), and three or fewer lymph nodes were retrieved in 60 of 76 (78.9%) versus 46 of 127 (36.2%). Overall, 90 patients (44.3%) underwent protocol‐adherent radical resection. Their characteristics by approach are shown in Table 2; the groups differed only in intraoperative transfusion, tumor size, and number of harvested lymph nodes.

**TABLE 2:** Clinicopathological characteristics of patients proven to be protocol‐adherent radical resection by operation method.

Operation method	Laparoscopic	Open	*p*
	(*N* = 14)	(*N* = 76)	
Age	66.1 ± 9.3	65.9 ± 9.9	0.943
Sex			
Male	5 (35.7%)	1.000	
Preop CA19‐9	10.8 ± 11.2	56.2 ± 138.3	0.007
Liver resection			
Wedge	7 (50.0%)	45 (59.2%)	0.566
IVb/V	7 (50.0%)	31 (40.8%)	
OP time	160.4 ± 128.6	198.6 ± 93.8	0.191
Estimated blood loss (mL)	461.1 ± 560.7	451.5 ± 473.6	0.946
IntraOP transfusion			
Yes	8 (57.1%)	15 (19.7%)	0.009
Tumor size	2.5 ± 1.6	4.2 ± 2.2	0.006
OP time	160.4 ± 128.6	198.6 ± 93.8	0.191
Number of harvested lymph nodes	8.1 ± 1.9	11.0 ± 5.7	0.001
Differentiation			
Well	1 (7.1%)		
Moderate	8 (57.1%)		
Poor	2 (14.3%)	0.345	
Perineural invasion			
Yes	2 (14.3%)	0.288	
Lymphovascular invasion			
Yes	2 (14.3%)	0.055	
T stage^a^			
T2a	6 (43%)	27 (35.5%)	
T2b	3 (21%)	22 (29%)	
T3	3 (21%)	27 (35.5%)	0.733
N stage			
N0	10 (71.4%)	35 (46.1%)	
N1	3 (21.4%)	26 (34.2%)	
N2	1 (7.1%)	15 (19.7%)	0.262
Adjuvant therapy			
Yes	7 (50.0%)	51 (67.1%)	0.355
Recurrence			
Yes	5 (35.7%)	0.294	

^a^ Two tumor location was unavailable due to previous cholecystectomy at other institution.

### 3.2 Recurrence

The median follow‐up duration for all patients was 35 months (range, 1–168 months). Tumor recurrence occurred in 108 patients (53%). Among patients who achieved protocol‐adherent radical resection, 39 patients (43%) experienced tumor recurrence, including 5 of 14 patients (35.7%) in the laparoscopic group and 34 of 76 patients (45.9%) in the open group.

Among patients who underwent laparoscopic surgery (*N* = 76), local recurrence occurred in 20 patients (20/76, 26.3%), systemic recurrence in 19 patients (19/76, 25.0%), and combined or other types of recurrence in 3 patients. In the subset of patients who underwent protocol‐adherent radical resection via laparoscopy (*N* = 14), local recurrence was observed in 2 patients (2/14, 14.3%), and systemic recurrence occurred in 3 patients (3/14, 21.4%).

Among patients who underwent open surgery (*N* = 127), local recurrence occurred in 31 patients (31/127, 24.4%), systemic recurrence in 28 patients (28/127, 22.0%), and combined or other recurrences in 5 patients. In patients who achieved protocol‐adherent radical resection with the open approach (*N* = 76), local recurrence occurred in 15 patients (15/76, 19.7%), systemic recurrence in 16 patients (16/76, 21.1%), and combined recurrence in 1 patient.

### 3.3 Survival by Operative Procedure

The 5‐year OS rate for all patients (*N* = 203) was 42.7%. The 5‐year OS rates for patients who achieved protocol‐adherent radical resection (*N* = 90) and those who underwent nonradical resection (*N* = 113) were 54.5% and 33.6%, respectively (*p* = 0.005, Figure 2). Subsequent survival analyses were performed exclusively in patients who achieved protocol‐adherent radical resection.

**FIGURE 2:**
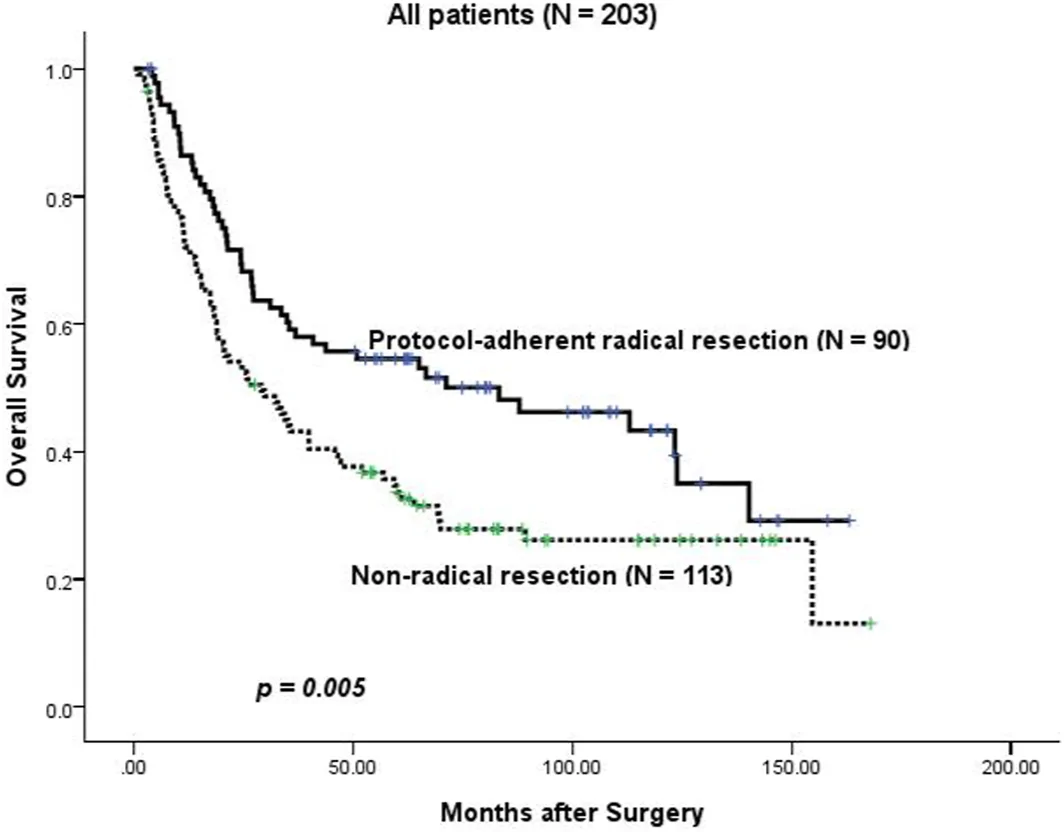
Overall survival rates of all patients. The 5‐year OS rates for patients who achieved protocol‐adherent radical resection (*N* = 90) was 54.5% and those who underwent nonradical resection (*N* = 113) was 33.6% (*p* = 0.005).

Among the 90 patients who achieved protocol‐adherent radical resection, the 5‐year OS rate was 61.5% in the laparoscopic group (*N* = 14) and 53.3% in the open group (*N* = 76; *p* = 0.288; Figure 3). Because only 14 laparoscopic patients met the radical criteria, this within‐subgroup comparison is underpowered and is therefore reported as exploratory and hypothesis‐generating rather than confirmatory.

**FIGURE 3:**
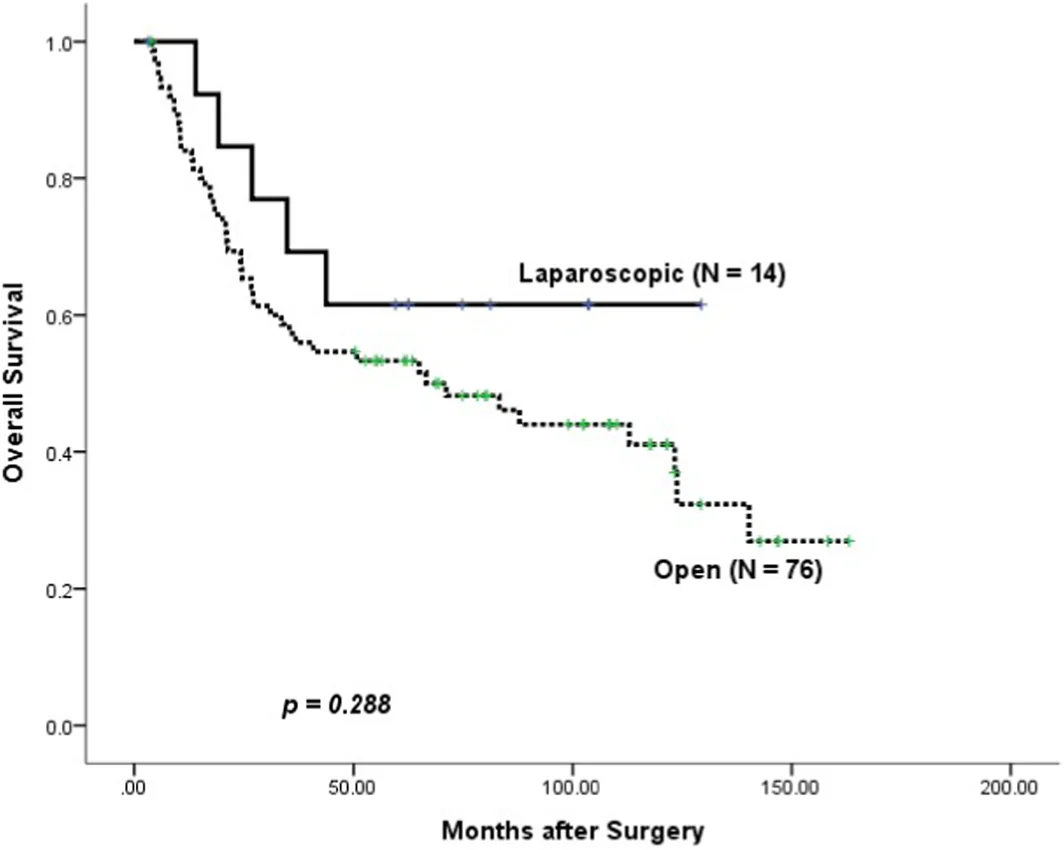
Overall survival rates of patients who achieved protocol‐adherent radical resection. The 5‐year OS rates for patients underwent laparoscopic surgery was 61.5% and that for patients underwent open surgery was 53.3% (*p* = 0.288).

The survival curves stratified between surgical approach and T stage or N stage were as follows: According to T stage, the 5‐year OS rates were 71.9% for patients with T2a GB cancer (*N* = 33), 65.4% for those with T2b GB cancer (*N* = 27), and 26.7% for those with T3 GB cancer (*N* = 30; *p* < 0.001; Supporting Information S1). Based on nodal status, the 5‐year OS rates were 74.9% for patients with N0 disease (*N* = 45), 41.4% for those with N1 disease (*N* = 29), and 20.0% for those with N2 disease (*N* = 16; *p* = 0.001; Supporting Information S1).

Because the approach was not randomised, the groups differed at baseline: laparoscopic patients were older with smaller tumors and less advanced T stage (standardised mean differences up to 0.52). After 1:1 propensity score matching, 54 balanced pairs were generated, with all standardised mean differences below 0.1. In the matched cohort, the 5‐year OS did not differ between approaches (46.1% vs. 49.9%; log‐rank *p* = 0.667; hazard ratio 0.90, 95% CI 0.55–1.47; Figure 4). The results were unchanged when N stage was added to the matching model (55 pairs; hazard ratio 1.05, 95% CI 0.64–1.73) and when the Cox model was adjusted for institution (hazard ratio 0.74, 95% CI 0.41–1.32). Notably, the disparity in oncological adequacy persisted after matching: adequate lymphadenectomy (≥ 4 nodes) in 22.2% of laparoscopic versus 74.1% of open resections, and protocol‐adherent radical resection in 20.4% versus 70.4%.

**FIGURE 4:**
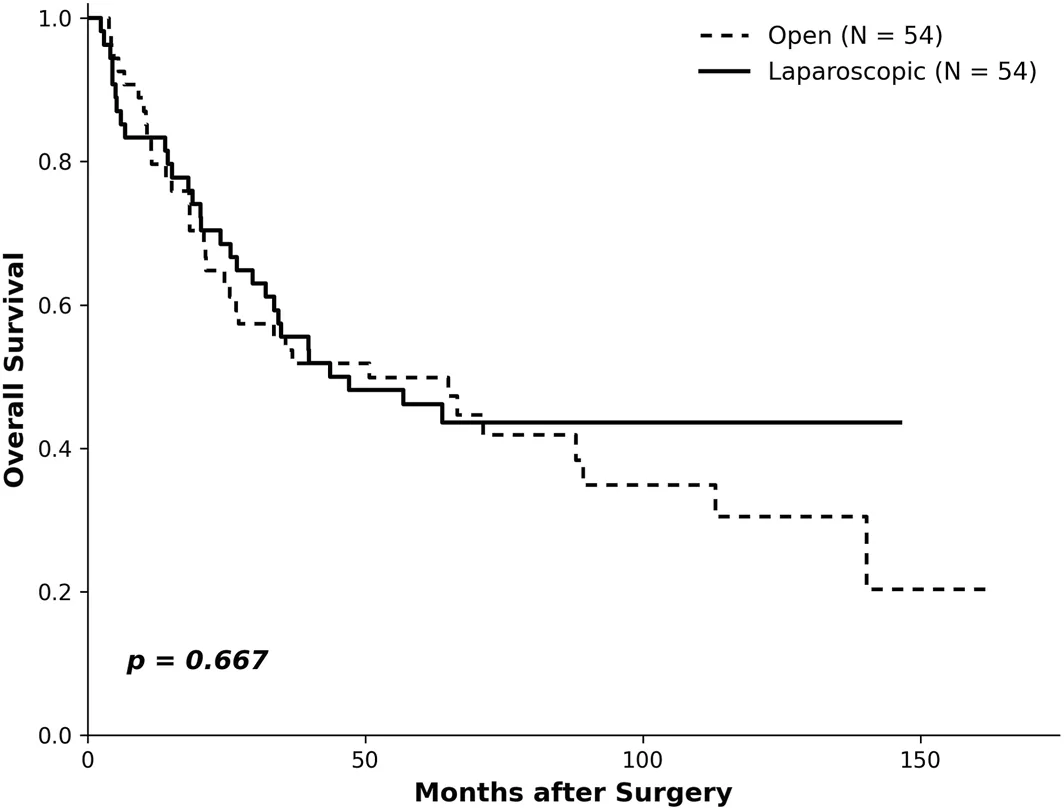
Overall survival rates of patients after propensity score matching based on surgical method. The 5‐year OS rates for patients underwent laparoscopic surgery was 46.1% and that for patients underwent open surgery was 49.9% (*p* = 0.667).

In the entire cohort (*N* = 203), subgroup analyses were conducted to evaluate the association between lymphovascular invasion and liver resection. Among patients with positive lymphovascular invasion, the 5‐year OS rate was 27.4% in those who underwent liver resection, compared with 13.3% in those who did not undergo liver resection (*p* = 0.122; Supporting Information S1). In contrast, among patients without lymphovascular invasion, the 5‐year OS rates were 62.3% for those who underwent liver resection and 30.1% for those who did not (*p* = 0.015; Supporting Information S1).

### 3.4 Prognostic Factors

In univariate analysis of patients who achieved protocol‐adherent radical resection (*N* = 90), AJCC T stage, N stage, intraoperative transfusion, lymphovascular invasion, perineural invasion, and elevated CA 19‐9 levels were all significantly associated with OS (all *p* < 0.10). Multivariate analysis in this subgroup identified N stage and elevated CA 19‐9 levels as independent prognostic factors for OS (Table 3).

**TABLE 3:** Predictors of survival in patients (*N* = 90) proven to be treated radical.

	Univariate analysis (log‐rank *p* value)	Multivariate analysis (cox regression (*p*)	Hazard ratio, 95% CI
T stage (AJCC 8th.)	< 0.001	0.208	
N stage	0.001	0.023	0.132–0.920
Intraoperative transfusion	0.016	0.269	
Perineural invasion	0.001	0.089	
Lymphovascular invasion	0.018	0.980	
Increased CA 19‐9 level	0.002	0.037	0.239–0.958

In the analysis of all patients (*N* = 203), AJCC T stage, N stage, intraoperative transfusion, lymphovascular invasion, perineural invasion, histologic differentiation, treated radical versus nonradical resection, and elevated CA 19‐9 levels were significantly associated with OS in univariate analysis (all *p* < 0.10). Multivariate analysis demonstrated that AJCC T stage, N stage, protocol‐adherent radical resection, perineural invasion, and elevated CA 19‐9 levels were independent prognostic factors for OS (Supporting Information S2).

## 4 Discussion

In the present study, 90 of 203 patients (44.3%) achieved protocol‐adherent radical resection: 14 of 76 (18.4%) in the laparoscopic group versus 76 of 127 (59.8%) in the open group. The dominant unmet criterion after laparoscopy was an insufficient lymph node yield (60/76, 78.9%), with liver resection omitted in 22 patients (28.9%). Patients achieving protocol‐adherent radical resection had significantly better 5‐year OS than those undergoing nonradical resection (54.5% vs. 33.6%; *p* = 0.005), whereas survival did not differ by surgical approach, either within the radical subgroup (*p* = 0.288) or after propensity score matching of the whole cohort (hazard ratio 0.90, 95% CI 0.55–1.47). Although the prognostic significance of N stage, CA 19–9 and perineural invasion is well established and we claim no novelty in this respect, these findings validate the internal consistency of our cohort. The distinct contribution of this study is methodological: to our knowledge it is the first to apply a per‐protocol (quality‐control) framework to laparoscopic radical resection for GB cancer, showing that true radical criteria were met in only 18.4% of laparoscopic operations and identifying inadequate lymphadenectomy as the dominant failure mode.

Several obstacles remain in laparoscopic radical resection for GB cancer: bile spillage from intraoperative GB injury, port‐site recurrence, and the technical demands of liver bed resection and lymphadenectomy around the hepatoduodenal ligament and posterior superior pancreatoduodenal area. Laparoscopic IVb/V segmentectomy requires meticulous parenchymal dissection to avoid bleeding from hepatic pedicles and veins, whereas nodal clearance demands isolation of the common bile duct, hepatic artery and portal vein without injury, together with duodenal mobilization (Kocher maneuver) to reach the posterior superior pancreatoduodenal nodes. Laparoscopic lymphadenectomy for GB cancer is therefore technically demanding even for surgeons experienced in laparoscopic pancreaticoduodenectomy, and this difficulty plausibly underlies the low nodal yield we observed.

Previous studies have reported that laparoscopic surgery for GB cancer yields acceptable short‐term outcomes, including intraoperative blood loss and bile leakage, length of hospital stay, surgical mortality, R1 resection rates, port‐site recurrence rates, and peritoneal recurrence rates [8, 9, 11, 12, 15, 16]. A limited number of studies evaluating oncological outcomes have suggested that laparoscopic re‐resection for selected patients with incidental GB cancer is oncologically noninferior to the open approach [6, 17, 18]. However, in most published series, it is unclear whether laparoscopic resections met the criteria defining radical surgery, and analyses were therefore generally based on an intention‐to‐treat principle. In contrast, the present study employed a per‐protocol analysis, classifying procedures as protocol‐adherent radical resection only when the predefined criteria were fulfilled.

Using the National Cancer Database, Hamad et al. found a median lymph node yield of 3 (interquartile range 1–6) for both approaches, and a similar proportion of patients with at least six nodes examined (open 29.3% vs. laparoscopic 25.4%; p = 0.10) [19]. Other series likewise report comparable nodal evaluation irrespective of approach [8, 16, 19, 20]. Among our patients achieving protocol‐adherent radical resection, the mean nodal yield was 8.1 ± 1.9 for the laparoscopic and 11.0 ± 5.7 for the open approach (*p* = 0.001).

Long‐term oncological outcomes following laparoscopic radical resection and open resection for GB cancer have been reported to be comparable in several studies [5, 6, 8, 11, 15, 16, 17, 18, 21, 22], and the findings of the present study were consistent with these reports.

Historically, hepatic progression of GB cancer was thought to occur via gallbladder venous drainage into the intrahepatic portal veins of segments IVb and V, based on computed tomography analyses, thereby supporting resection of these segments to remove potential hepatic spread [23, 24, 25]. More recent studies, however, have demonstrated that direct liver invasion and portal tract invasion represent the principal modes of hepatic spread in resectable GB cancer [3, 26, 27, 28]. Most studies have reported similar postoperative survival outcomes between patients undergoing GB bed resection and those undergoing resection of liver segments IVb and V. Accordingly, the IHPBA‐APHPBA clinical guidelines recommend liver resection—either wedge resection of the GB bed or segmentectomy of IVb/V—when necessary to achieve a negative surgical margin, a recommendation that is further supported by the findings of the present study.

Lymphovascular invasion may represent a potential mechanism for the hepatic spread of tumor cells, and the present study suggests that this factor may partly explain the observed discrepancy in survival rates between patients who underwent liver resection and those who did not. Future studies focusing on the interaction between liver resection and lymphovascular invasion are warranted to refine further the indications and extent of hepatic resection in GB cancer surgery.

This study has several limitations. First, its retrospective design carries an inherent risk of selection bias: the approach reflected surgeon preference, and the open group harbored more advanced disease. Propensity score matching reduced measured confounding, but residual and unmeasured confounding cannot be excluded. Second, the comparison within the protocol‐adherent radical subgroup rested on only 14 laparoscopic patients; it is underpowered and matching within this subgroup was not feasible, so it must be regarded as exploratory and hypothesis‐generating. Third, institution could not be entered into the matching model because one center performed only open resections; an institutional effect therefore cannot be fully separated from that of the approach, although adjusting the Cox model for institution within the matched cohort left the conclusion unchanged (hazard ratio 0.74, 95% CI 0.41–1.32). Fourth, 10 patients with unrecorded tumor size were excluded from the matched analysis. Nevertheless, the long follow‐up and the per‐protocol design partially mitigate these limitations. To our knowledge this is the first study to apply per‐protocol quality control to laparoscopic radical resection for GB cancer. Larger, ideally randomized, studies are needed.

In conclusion, regardless of whether a laparoscopic or open approach is used, radical surgery—including appropriate hepatic resection with the retrieval of at least four lymph nodes—is essential for improving long‐term prognosis in patients with GB cancer. N stage and elevated CA 19‐9 levels were independently associated with OS.

## Author Contributions

**Tae Ho Hong:** writing – original draft, data curation, investigation, formal analysis. **Kwang Yeol Paik:** conceptualization, writing – review and editing, supervision. **Yoon kyung Woo:** methodology, data curation, investigation, resources. **Jae Hyun Han:** methodology, data curation, investigation. **Jung Woo Lee:** conceptualization, methodology, data curation, investigation, validation. **Jong Woo Lee:** investigation, data curation, validation. **Dong Do You:** conceptualization, funding acquisition, project administration, formal analysis, validation.

## Funding

This research was supported by a National Research Foundation of Korea (NRF) grant funded by the Korean government (no. 2018R1D1A1B05049485).

## Conflicts of Interest

The authors declare no conflicts of interest.

## Supporting information


Supporting Information S1

Table S1

## Data Availability

The data that support the findings of this study are available on request from the corresponding author. The data are not publicly available due to privacy or ethical restrictions.
