# Supplementary material for: Per Protocol Analysis and Long‐Term Outcomes of Laparoscopic Radical Resection for Gallbladder Cancer Compared With Open Method
Source: World J Surg. 2026 Aug 24;50(10):3065–73. doi: 10.1002/wjs.70535 (PMC13662492; doi:10.1002/wjs.70535)
Supplement: Supplementary file 1 [file WJS-50-3065-s001.docx]

A

**T2a**

**T2b**

**T3**

***p* = 0.683**

***p* = 0.002**

A

Supplementary Figure 1. Overall survival rates patients who achieved protocol-adherent radical resection based on the T stage and N stage.

A, The 5-year OS rates were 71.9% for patients with T2a GB cancer, 65.4% for those with T2b GB cancer, and 26.7% for those with T3 GB cancer (*p* < 0.001).

B, The 5-year OS rates were 74.9% for patients with N0, 41.4% for those with N1 stage, and 20.0% for those with N2 stage (*p* = 0.001).

B

A

B

Supplementary Figure 2. Subgroup analysis to evaluate the association between liver resection and lymphovascular invasion. A, the 5-year OS rates for patients with positive lymphovascular invasion who were underwent liver resection was 27.4%; that for patients who did not underwent liver resection was 13.3% (*p* = 0.122). B, the 5-year OS rates for patients with negative lymphovascular invasion who underwent liver resection was 62.3%; that for patients who did not underwent liver resection was 30.1% (*p* = 0.015).
