# Supplementary material for: Per Protocol Analysis and Long‐Term Outcomes of Laparoscopic Radical Resection for Gallbladder Cancer Compared With Open Method
Source: World J Surg. 2026 Aug 24;50(10):3065–73. doi: 10.1002/wjs.70535 (PMC13662492; doi:10.1002/wjs.70535)
Supplement: Supplementary file 2 [file WJS-50-3065-s002.docx]

| Supplementary Table 1. Predictors of Survival in all Patients (N = 203) | | | |
| --- | --- | --- | --- |
|  | Univariate analysis (Log-Rank *p* value) | Multivariate analysis (Cox regression (*p*) | Hazard Ratio, 95% CI |
| T stage (AJCC 8th.) | < 0.001 | 0.030 | 0.279-0.917 |
| Histologic differentiation | < 0.001 | 0.153 |  |
| Intraoperative transfusion | 0.032 | 0.591 |  |
| Lymphovascular invasion | < 0.001 | 0.116 |  |
| Perineural invasion | < 0.001 | 0.003 | 0.635-3.886 |
| Treated Radical vs Non-radical | 0.004 | 0.001 | 0.298-0.721 |
| Increased CA 19-9 level | < 0.001 | 0.002 | 0.345-0.793 |
| Adjuvant treatment | 0.180 |  |  |
| N stage | < 0.001 | 0.013 | 0.167-0.883 |
